# Schultze's Method of Artificial Respiration

**Published:** 1899-07-22

**Authors:** 


### SCHULTZE'S METHOD OF ARTIFICIAL RESPIRATION.

At a recent meeting of the Royal Academy of Medicine in Ireland, Dr. Kidd, the President, read notes of a successful case of Cæsarian section. At the time of the operation the child, on being removed, was found to be partially asphyxiated, but under the care of Dr. Stevens, who Schultzed him, he came round, although he never cried lustily. On the third day he died. The previous day he had had a slight convulsion, and his muscles had continually seemed to be tense. He had also thrown up coffee-ground vomit. At the necropsy some slight intussusception of the small intestine was found, which had probably been caused during the death agony, and some hæmorrhage at the lower end of the œsophagus. The question arose as to whether the latter might have been the result of Schultzing, notwithstanding that it had been done by experienced hands. In regard to this, it was said that Sylvester's method was far more likely than Schultze's to cause hæmorrhage, which is probably true. But, in any case, the possibility of such an event happening is worth bearing in mind.

